# Ethnicity and Occupational Pension Membership in the UK

**DOI:** 10.1111/spol.12137

**Published:** 2015-04-14

**Authors:** Athina Vlachantoni, Zhixin Feng, Maria Evandrou, Jane Falkingham

**Affiliations:** ^1^ Centre for Research on Ageing and ESRC Centre for Population ChangeFaculty of Social and Human SciencesUniversity of SouthamptonSouthamptonUK

**Keywords:** Employment patterns, Pension protection, Ethnicity, UK Household Longitudinal Study

## Abstract

Reflecting a relatively low‐value Basic State Pension, occupational pensions have historically been a key aspect of pension protection within Britain. Existing research shows that minority ethnic groups are less likely to benefit from such pensions and are more likely to face poverty in later life, as a result of the interaction of their labour market participation and pension membership patterns. However, the lack of adequate data on ethnic minorities has so far prevented the direct comparison of different ethnic groups, as well as their comparison to the White British group. Using data from the UK Household Longitudinal Study, this article explores patterns of employment and the odds ratios of membership in an employer's pension scheme among working‐age individuals from minority ethnic groups and the White British population, taking into account factors not used by previous research, such as one's migration history and sector of employment (public/private). The analysis provides new empirical evidence confirming that ethnicity remains a strong determinant of one's pension protection prospects through being in paid work, being an employee and working for an employer who offers a pension scheme. However, once an individual is working for an employer offering a pension scheme, the effect of ethnicity on that person's odds of being a member of that scheme reduces, except among Pakistani and Bangladeshi individuals for whom the differentials remain. The article also provides evidence on the pension protection of Polish individuals, a relatively ‘new’ minority group in the UK.

## Introduction

Existing research has provided evidence of the differentials between ethnic groups in the UK in terms of labour market participation (Allmark *et al*. 2010), which, for some ethnic groups, has led to a disadvantage in terms of pension protection (Barnes and Taylor 2006). According to research by the Pensions Policy Institute, individuals from ethnic minorities ‘have many of the “alarm bell” characteristics that are associated with lower pension incomes’ (Steventon and Sanchez 2009), such as low earnings, breaks in their employment record and a high prevalence of self‐employment which can lead to pension insecurity. However, among existing research on the association between ethnicity and occupational pension membership which highlights the disadvantageous position of ethnic minorities (see Ginn and Arber 2001), key factors such as the migration history of individuals as well as the sector (public/private) in which they work have been relatively under‐researched. Previous research has shown that in the British context, occupational pensions can often make the difference between an individual facing a poverty risk in later life, or not (Bardasi and Jenkins 2010), and contributes to a body of work aimed at improving our understanding of the circumstances of future cohorts of individuals from minority ethnic groups (Lupton and Power 2004; Dale *et al*. 2006). The 2011 UK Census showed that individuals from minority ethnic groups comprised about 7.8 million of the total population in England and Wales (ONS 2012a), and it has been projected that there will be 3.8 million individuals from Black and minority ethnic (BME) groups aged 65 and over by 2051 (Lievesley 2010). This article updates and extends previous research by analyzing recently available data from Understanding Society to investigate ethnic disadvantage in pension protection. Novel aspects of the research include, first, the use of nested regressions starting from whether an individual is in paid work, then whether that person is an employee, then whether that person is working for an employer offering a pension scheme, and lastly whether that person is a member of such a scheme. Understanding this ‘sequence’ of differentials is an original part of our article. Second, the article provides empirical evidence based on recent data, when existing empirical research dates from the 1990s. Third, the analysis includes a separate Polish group which is a relatively ‘new’ minority group, compared with ethnic groups which started migrating to the UK in the 1950s, 1960s and 1970s (e.g. Black Caribbean, Pakistani, Bangladeshi, Indian). Lastly, the analysis includes additional factors which have not been taken into account in previous research (migration history, whether the individual works in the public or private sector).

## Differentials in Labour Market Participation among Minority Ethnic Groups in Britain

Over the last two decades or so, the academic literature has emphasized substantial differentials between BME groups and the White majority in terms of economic and social resources, as well as health status (Allmark *et al*. 2010; Becares *et al*. 2012; Smith *et al*. 2000; Evandrou 2000a, 2000b; Steventon and Sanchez 2009). Part of such differentials relate to patterns of labour market participation, with existing research showing that individuals from most BME groups are less likely than White British individuals to be in paid employment, and there are significant gender differences in particular groups. However, unravelling the diversity within the BME population as a whole, previous research has emphasized the need to distinguish between groups in a more or less advantageous position, as well as between the two genders. For example, research by Allmark *et al*. (2010) showed that about one‐third of Bangladeshi, Pakistani and Black Caribbean men aged between 25 and 64 were unemployed compared to 15 per cent of White men, and more than 80 per cent of Bangladeshi and Pakistani women in the same age group were unemployed compared to 30 per cent of White women. When in paid work, individuals from certain BME groups are more likely to work part‐time or to be self‐employed, and to have lower earnings than their White counterparts (PPI 2003). For example, about 28 per cent of Indians of working age were in a managerial or professional group compared to 14 per cent of Pakistanis and 11 per cent of Bangladeshis (ONS 2006).

The explanation put forward for such differences in the patterns of labour force participation is complex. Berthoud (1998) notes that reasons such as lower levels of educational qualifications, lower levels of fluency in English, cultural/religious values relating to the primacy of family care over paid work, or the fact that particular ‘waves’ of migration were associated with particular (low‐paid) sectors of the labour market, may contribute to such differences. Evidence from nationally representative sources and the Census display stark contrasts in these respects. In 2004, one‐quarter of Indians held a degree qualification compared with 12 per cent of Pakistanis and 8 per cent of Bangladeshis (ONS 2006). However, research comparing the circumstances of different cohorts of BME groups has identified changing attitudes towards paid work and family formation, with second‐generation migrants who were educated in the UK being more likely to set up smaller families and dual‐earner households (Barnes and Taylor 2006).

Such differentials in the likelihood of being in paid work among ethnic groups tend to both persist and accumulate over the life course, resulting in a higher poverty risk for older individuals from particular ethnic groups (Ginn and Arber 2001). However, such poverty risk to some extent is accentuated by particular characteristics relating to the health profiles, living arrangements and cultural norms of certain groups. For instance, Berthoud's (1998) study of the incomes of BME groups noted that it was the *combination* of Pakistani and Bangladeshi men's and women's lower chances of being in employment and earning sufficient earnings *with* the relatively high number of persons living in their household, which translate into a higher risk of poverty and a higher reliance on the welfare state. In 2001, the average household size among Bangladeshis was 4.5 persons, followed by 4.1 among Pakistanis and 3.3 among Indians (ONS 2006), while 44 per cent of Bangladeshi households were overcrowded compared with 6 per cent of households among the White British majority (ONS 2006). Similarly, Evandrou (2000b) has used data from the General Household Survey to show that Bangladeshi men and women at every age from 16 years and over are more likely than individuals from other BME groups and from the White British majority, to report a limiting long‐standing illness.

## Ethnic Minorities in the British Pension System

Since the 1940s, when its foundations were laid, the modern British pension system has been characterized by a contributory flat‐rate state pension paid at a relatively low level (approximately 16 per cent of National Average Earnings in 2009) and a relatively small public earnings‐related scheme, topped up by means‐tested benefits for those on low incomes and by private (non‐state) pensions for those with middle and high incomes (Evandrou and Falkingham 2009; PPI 2013). Two key principles permeating the British pension system, those of a close link between employment records and of the concept of a ‘top‐up’ in addition to first‐ and second‐tier protection from the state, have exposed the system's inability to cater for particular social groups with employment records not ‘fitting’ with eligibility rules, who are likely to face a higher poverty risk in later life as a result, such as women, disabled persons and persons from ethnic minority groups (Pemberton *et al*. 2006). Against this background, the importance of (non‐state) occupational pensions in shielding individuals from poverty in later life has been emphasized in the academic literature (Bardasi and Jenkins 2010).

Recent policy reforms have sought to close the coverage gap and to contribute to pension adequacy for particular groups of the population, such as those on low incomes. For example, individuals who reach the State Pensionable Age from 2016, and have acquired 35 years of contributions, will receive the single‐tier State Pension, while those with fewer years of contributions will receive a pro‐rata amount. In terms of private (non‐state) pension contribution, the Pensions Act 2008 introduced auto‐enrolment into a defined contribution pension scheme for all employees with sufficient earnings and aged between 22 and the state pension age (SPA) entering the labour market from October 2012. Large (with 250 or more employees), medium (between 50 and 249 employees) and small (up to 49 employees) companies are being gradually introduced in the system between 2012 and 2017. However, there exist concerns about the extent to which individuals from particular social groups can or have benefited from such reforms.

Differentials between BME groups and the White majority relating to the level and nature of participation in the labour market can result in differentials in terms of occupational pension membership, and such pension membership can provide the most important shield against poverty in later life in the British pension context (Bardasi and Jenkins 2010). Research by Nesbitt and Neary (2001) found that Pakistani and Bangladeshi individuals were less likely than White individuals to contribute to, or to be aware of, an employer's pension scheme. Ginn's and Arber's (2001) work on the ‘ethnic pension penalty’ emphasized the role of gender in exacerbating the private pension protection gap, particularly for Bangladeshi and Pakistani women, and noted that controlling for labour market participation alone did not fully explain differentials in pension protection between ethnic minority groups. More recent qualitative research commissioned by the Department for Work and Pensions, involving interviews with individuals from the Indian, Pakistani, Black Caribbean, Black African, Bangladeshi and Chinese groups, reflected limited knowledge of pensions (Barnes and Taylor 2006). Similar results regarding a poor understanding of pension protection among ethnic minority groups were found by Gough and Hick (2009), whose qualitative interviews revealed scepticism among respondents about the ability to continue to afford contributions while on low earnings, as well as intentions of future return migration posing disincentives to invest in retirement planning. Other research has noted that ethnicity is only one of a number of characteristics, including age, gender, education and social class, which affect individuals' understanding of financial products and their ability to make long‐term decisions (Vickerstaff *et al*. 2012).

In order to improve and update our understanding of such differentials, this article reports on analysis of the association between ethnicity and a working‐age individual's chances of being a member of an occupational pension scheme, taking into account additional factors not incorporated in previous research, such as individuals' migration history and the occupational sector in which they work. In trying to unravel this association, the analysis also focuses on analyzing the association between ethnicity and the three preceding stages of being in paid work (or not); being an employee (or self‐employed); and working as an employee for an employer who offers a pension scheme (or not).

## Data and Methodology

The analysis uses data from wave 1 of the UK Household Longitudinal Study (UKHLS) (collected between 2009 and 2011), which is a longitudinal survey of the members of approximately 40,000 households in the UK. The dataset is ideal for this study, as it includes an Ethnic Minority Boost Sample, designed to provide at least 1,000 individuals from five ethnic groups: Indian, Pakistani, Bangladeshi, Caribbean and African. In addition, the dataset allows us to examine the pension prospects of a relatively ‘new’ minority group, that of Polish individuals, comparing their situation with that of individuals from more ‘traditional’ ethnic groups, such as the Indian and Pakistani groups. The analytical sample for this article includes all adults aged between 25 and the SPA (60 for women, 65 for men at the time of data collection), totalling 30,427 respondents, of whom 4,996 came from the five ethnic groups above.

## Independent Variables

*Ethnicity* was recoded into the following groups, which include the five groups for which cell counts have been boosted in the UKHLS: White British, Polish, Other White, Mixed, Indian, Pakistani, Bangladeshi, Other Asian, Caribbean, African, and Other ethnic groups.^1^ The Polish group has been distinguished in the sample as an example of a ‘new’ minority group in the UK, having migrated into the country more recently, relative to groups such as the Indian, Pakistani and Bangladeshi groups, and for which little research exists in the area of pension protection. In 2010, there were over 530,000 Polish‐born individuals in the UK, 86 per cent of whom were aged between 16 and 64 (ONS 2011).

*Age* was recoded into five‐year groups starting at 25 and ending at 59 (representing women's SPA) and 64 (representing men's SPA), although current government policies are in the process of increasing the SPA for both genders into the future.

*Sex* is included in the analysis in order to observe its independent effect on an individual's chances in terms of labour market participation and occupational pension membership. However, the analysis was also run separately for men and women in order to unravel gender differences.

*Marital status* was recoded into the following categories: single never married; married or cohabiting; divorced or separated; widowed.

The variables of whether someone *Cares for a ‘handicapped'/other person in household* and whether someone has *Children living in the household* (none; children under 5; children above 5), allowed us to investigate the impact of informal care provision on an individual's chances of being in paid work.

*Self‐reported general health* was recoded into three categories: excellent/very good/good; fair; poor.

Two variables indicating the report of a *limiting long‐standing illness* were recoded to derive a new variable with four categories: no longstanding illness; longstanding illness but not limiting; longstanding illness and limiting; no longstanding illness but reports limitations.

*Highest educational qualification* 2 was recoded into six categories: degree; other higher qualification; A‐levels; GCSE; other qualification (achieved outside the UK); no qualifications.

*Housing tenure* was recoded into seven categories: own outright; own with mortgage; rented from local authority; rented from Housing Association; rented from employer; rented privately; and other (e.g. living rent‐free or squatting).

*Occupational social class* was recoded into four categories: management and professional; intermediate; lower supervisory and technical; and semi‐routine, routine and never worked/long‐term unemployed. An individual's *occupational sector* reflected whether that person worked in the public or private sector.

*Quintile of take‐home pay* allows us to explore the position of individuals in the distribution of the population aged between 25 and 59/64, and ranges from the 1st (poorest) to the 5th (richest) quintile.

The *migrant history* variable allows us to examine the impact of the timing of migrants' move to Britain, compared to non‐migrants. This variable distinguished between three categories: those who were born in Britain to British parents (non‐migrants); those who were born abroad but who migrated to Britain (first‐generation migrants); and those who were born in Britain to migrant parents (second‐generation migrants). This variable is a proxy for a number of crucial influences on an individual's employment patterns, such as their language skills and cultural influences.

## Dependent Variables

**Figure 1:**
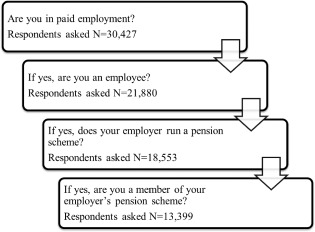
Questions used in the analysis *Source*: UKHLS, data from 2009 to 2011.

The dependent variables use four questions in the UKHLS which are asked in sequence, and the final question asks respondents whether they are members of their employer's pension scheme. The sequence of the questions used for this analysis is shown in figure 1.

Tables 1 and 2 present descriptive results on the employment patterns and employer's pension membership of working‐age individuals from different ethnic groups and by gender. Table 1 shows that, among men, the Polish (92 per cent) and Indian (86 per cent) groups are more likely to be in paid work than the White British group, while Caribbean men are the least likely (68 per cent). Among women of working age, it is the Polish (79 per cent) followed by the White British (74 per cent) groups that are the most likely to be in paid work, while Pakistani and Bangladeshi women are significantly less likely to be in paid work (both 30 per cent). Once they are in work, 80 per cent or more of men from most ethnic groups work as employees, however that percentage is 70 per cent among Pakistani men. Among women who are in paid work, the percentage who are employees is around or above 90 per cent for most ethnic groups, except for the group of Other Ethnicities, where 84 per cent of women are employees.

**Table 1:** Percentage of individuals aged between 25 to SPA being in paid work and of those, being an employee, by ethnic group and gender

Ethnic group	Being in paid work						Being an employee					
	**Men**			**Women**			**Men**			**Women**		
	*Yes*	*No*	*Total*	*Yes*	*No*	*Total*	*Yes*	*No*	*Total*	*Yes*	*No*	*Total*
White British	79.1%	20.9%	100% N = 10,196	74%	26%	100% N = 11,847	81.5%	18.5%	100% N = 7,895	91.4%	8.6%	100% N = 8,668
Polish	91.7%	8.3%	100% N = 109	79.2%	20.8%	100% N = 143	79.7%	20.3%	100% N = 98	89%	11%	100% N = 112
Other White	76.6%	23.4%	100% N = 579	73.9%	26.1%	100% N = 739	80.8%	19.2%	100% N = 434	87.4%	12.6%	100% N = 527
Mixed	76.2%	23.8%	100% N = 203	66.7%	33.3%	100% N = 326	80.6%	19.4%	100% N = 138	92.9%	7.1%	100% N = 200
Indian	85.8%	14.2%	100% N = 682	66%	34%	100% N = 656	82.6%	17.4%	100% N = 565	93%	7%	100% N = 409
Pakistani	79%	21%	100% N = 470	29.9%	70.1%	100% N = 552	69.2%	30.8%	100% N = 362	89.1%	10.9%	100% N = 145
Bangladeshi	75%	25%	100% N = 399	30.2%	69.8%	100% N = 382	78.7%	21.3%	100% N = 261	94.4%	5.6%	100% N = 92
Other Asian	79.8%	20.2%	100% N = 294	61%	39%	100% N = 361	81.8%	18.9%	100% N = 225	89.5%	10.5%	100% N = 207
Caribbean	67.5%	32.5%	100% N = 304	69.9%	30.1%	100% N = 481	81.2%	18.8%	100% N = 192	92.5%	7.5%	100% N = 329
African	70.6%	29.4%	100% N = 446	58.5%	41.5%	100% N = 624	87.8%	12.2%	100% N = 302	96.2%	3.8%	100% N = 339
Other ethnic groups	69.4%	30.6%	100% N = 301	58%	42%	100% N = 333	82%	18%	100% N = 201	83.7%	16.3%	100% N = 179

*Note*: Weighted percentages, unweighted sample counts.

**Table 2:** Percentage of employee individuals aged between 25 to SPA working for an employer who offers a pension scheme and of those, being a member of that scheme, by ethnic group and gender

Ethnic group	Working for an employer who offers a pension scheme						Being a member of the employer's pension scheme					
	**Men**			**Women**			**Men**			**Women**		
	*Yes*	*No*	*Total*	*Yes*	*No*	*Total*	*Yes*	*No*	*Total*	*Yes*	*No*	*Total*
White British	74.8%	25.2%	100% N = 6,331	75.3%	24.7%	100% N = 7,803	76.2%	23.8%	100% N = 4,743	75.6%	24.4%	100% N = 5,846
Polish	48.5%	51.5%	100% N = 75	46.7%	53.3%	100% N = 88	42.6%	57.4%	100% N = 37	48.8%	51.2%	100% N = 39
Other White	67.3%	32.7%	100% N = 346	69.2%	30.8%	100% N = 454	69.1%	30.9%	100% N = 229	75.4%	24.6%	100% N = 314
Mixed	64.4%	35.6%	100% N = 109	68.9%	31.1%	100% N = 183	68.1%	31.9%	100% N = 76	79%	21%	100% N = 135
Indian	64.2%	35.8%	100% N = 458	63.4%	36.6%	100% N = 371	66.8%	33.2%	100% N = 276	73.2%	26.8%	100% N = 235
Pakistani	54%	46%	100% N = 249	72.3%	27.7%	100% N = 124	60%	40%	100% N = 130	57.6%	42.4%	100% N = 86
Bangladeshi	44.6%	55.4%	100% N = 200	64.7%	35.3%	100% N = 85	73.1%	26.9%	100% N = 72	45.5%	54.5%	100% N = 58
Other Asian	63.7%	36.3%	100% N = 180	62.6%	37.4%	100% N = 182	64.8%	35.2%	100% N = 109	63.8%	36.2%	100% N = 119
Caribbean	67.6%	32.4%	100% N = 154	80.2%	19.8%	100% N = 299	71.7%	28.3%	100% N = 103	75.3%	24.7%	100% N = 238
African	66.7%	33.3%	100% N = 249	66.4%	33.6%	100% N = 312	61%	39%	100% N = 159	65.8%	34.2%	100% N = 206
Other ethnic groups	61.6%	38.4%	100% N = 160	70%	30%	100% N = 148	61.3%	38.7%	100% N = 97	66.1%	33.9%	100% N = 96

*Note*: Weighted percentages, unweighted sample counts.

Table 2 turns to examine the percentage among men and women employees who work for an employer offering a pension scheme and the percentage who are members of such a scheme. The table shows that White British men are the most likely to work for an employer who offers a pension scheme (75 per cent), while Pakistani (45 per cent), Polish (49 per cent) and Bangladeshi (54 per cent) men are the least likely. Among women also significant differentials exist, with Caribbean (80 per cent) women being the most likely to work for an employer offering a pension scheme, and Polish (47 per cent) women being the least likely. Lastly, among men who work for an employer offering a pension scheme, the White British men (76 per cent) are the most likely to be members of such a scheme, while Polish (43 per cent), Pakistani (60 per cent) and African (61 per cent) men are the least likely. Among women it is the Mixed group (79 per cent) who are the most likely to be members of their employer's pension scheme, while Bangladeshi (46 per cent), Polish (49 per cent) and Pakistani (58 per cent) women are the least likely. Such results indicate three distinct points: first, there are significant differentials between the White British majority group and BME groups in terms of labour market participation and employer's pension protection; second, there exists considerable diversity within the BME group in these aspects, highlighting disadvantages between different ethnic groups and pointing to the Bangladeshi and Pakistani group (and women in particular) as being the most disadvantaged in terms of the labour market; and, third, there are significant gender differences throughout the groups.

In addition, we explored the differentials between ethnic groups in terms of whether individuals work in the public or private sector, and found that Caribbeans are the most likely to work in the public sector (49 per cent of this group), followed by the Mixed (44 per cent), African (42 per cent) and White British (41 per cent), while the Polish (15 per cent) were the least likely, and all other ethnic groups showed percentages between 30 per cent and 35 per cent.

## Effect of Ethnicity on One's Chances of Being in Paid Work

Table 3 shows that, for both men and women, a combination of demographic, health and socio‐economic characteristics are associated with being in paid employment or not, and ethnicity has a strong effect on this relationship. Being under the age of 50, with high educational qualifications and owning one's home with a mortgage are strongly associated with one's chances of being in paid employment, while being female and single never married reduce one's odds of being in paid employment. The report of excellent, good or very good health, and no report of a long‐standing illness are also positively associated with being in paid work, and the negative impact of caring for someone in the household or having children (of any age) is also reflected in Model 1. Among ethnic minority groups, Polish individuals are the only group who are more likely to be in paid work than the White British majority, while all other groups are less likely. For example, the odds of being in paid work among Pakistani and Bangladeshi individuals are 0.3 and 0.4 the odds among White British individuals, respectively. When the analysis was run separately for men and women (Models 2 and 3), the results are broadly similar, although certain differences are reflected. Being separated or divorced is associated with higher odds of being in paid work among women, but with lower odds among men; and the single never married category had lower odds of employment for men but not women, while having children (of any age) is associated with lower odds of being in paid work for women, but is not a significant factor for men's employment. Renting from one's employer is associated with higher odds of being in paid work for men, but is not a significant factor for women's employment. Lastly, being Polish is strongly associated with higher odds of being in paid work for men, but is not a significant factor in the women's model, while the (negative) impact of coming from an ethnic minority group is accentuated for women except for Caribbean and Polish women. For example, among Pakistani and Bangladeshi women, the odds of being in paid work are 0.15, and 0.22 among White British women, respectively.

**Table 3:** Odds ratios of being in paid employment

		Both genders	Men	Women
	*% of individuals in each group*	*Odds ratios*	*Odds ratios*	*Odds ratios*
In paid employment (N = 21,895)	72.00%			
Not in paid employment (N = 8,532)	28.00%			
**Age**				
25–29 (ref)	13.00%	1	1	1
30–34	13.70%	1.27***	1.38**	1.16*
35–39	14.90%	1.26***	1.33**	1.1
40–44	15.40%	1.36***	1.32**	1.19*
45–49	14.20%	1.34***	1.17	1.18
50–54	12.50%	1.15*	0.93	1.04
55–59	11.10%	0.78***	0.68***	0.69***
60–64	5.30%	0.23***	0.24***	ns
**Gender**				
Male (ref)	46.00%	1	ns	ns
Female	54.00%	0.50***	ns	ns
**Marital status**				
Married (ref)	57.50%	1	1	1
Single never married	26.90%	0.78***	0.54***	0.98
Separated/divorced	14.10%	1.03	0.70***	1.28***
Widowed	1.50%	0.87	0.63*	0.88
**Education**				
Degree (ref)	27.60%	1	1	1
Other high	10.60%	0.96	0.94	0.95
A level	9.40%	0.73***	0.96	0.65***
GCSE	22.50%	0.65***	0.81**	0.59***
Other qualification	9.10%	0.65***	0.96	0.50***
No qualifications	20.70%	0.41***	0.60***	0.31***
**Housing tenure**				
Own outright (ref)	17.60%	1	1	1
Own with mortgage	47.20%	2.28***	2.49***	2.28***
Rented from local authority	11.10%	0.46***	0.38***	0.49***
Rented from Housing Association	7.00%	0.49***	0.37***	0.55***
Rented from employer	1.00%	1.92***	5.04***	1.09
Rented privately	15.60%	0.82***	0.79**	0.79**
Other	0.30%	0.86	0.85	0.86
**Ethnicity**				
White British (ref)	72.30%	1	1	1
Polish	0.80%	1.59*	2.04*	1.4
Other White	4.30%	0.70***	0.68*	0.72***
Mixed	1.70%	0.61***	0.49***	0.68**
Indian	4.40%	0.60***	0.81	0.50***
Pakistani	3.40%	0.30***	0.65***	0.15***
Bangladeshi	2.60%	0.36***	0.50***	0.22***
Other Asian	2.20%	0.42***	0.52***	0.38***
Caribbean	2.60%	0.83*	0.53***	1.07
African	3.50%	0.59***	0.48***	0.68***
Other ethnic	2.10%	0.46***	0.46***	0.47***
**Self‐rated general health**				
Excellent/good/very good (ref)	80.70%	1	1	1
Fair	13.00%	0.62***	0.60***	0.64***
Poor	6.30%	0.19***	0.17***	0.20***
**Limiting longstanding Illness**				
No longstanding illness (ref)	50.90%	1	1	1
Longstanding illness but not limiting	10.20%	0.84**	0.74***	0.94
Longstanding illness and limiting	21.80%	0.50***	0.43***	0.54***
No longstanding illness but reports limitations	16.90%	0.92+	0.97	0.93
**Cares for handicapped/other in household**				
Yes (ref)	6.20%	1	1	1
Missing	12.40%	1.73***	1.82***	2.25***
No	81.30%	2.23***	2.07***	2.36***
**Children**				
None (ref)	54.80%	1	1	1
Children under 5	23.30%	0.46***	0.96	0.28***
Children above 5	21.80%	0.71***	0.94	0.57***
**Constant**		5.77***	5.26***	3.67***

*Notes*: N = 30,427, *** p < 0.001; ** p < 0.01; * p < 0.05; ^+^ p < 0.1.

## Association between Ethnicity and One's Chances of Being an Employee

Among those who were in paid work, table 4 shows the odds ratios of being an employee, as opposed to being self‐employed. Model 1 shows that young age (aged between 25 and 29) and being female, are positively associated with being an employee, as are renting one's house from a local authority, Housing Association or privately, or owning one's home with a mortgage. The impact of ethnicity on being an employee is only strong for some of the groups, with the Pakistani group being less likely than the White British group to be employees, but the Indian and African groups being more likely to be employees. Lastly, there is little difference between being a first‐ or second‐generation migrant on one's odds of being an employee, with the odds of being an employee among either a first‐ or second‐generation migrants being 0.76–0.79 of the odds among non‐migrants (reference category, 1.00). Examining the odds ratios of being an employee separately for men and women (Models 2 and 3) showed that education had a differential effect, with lower‐level education (GCSE to A‐levels) being associated with higher odds of being an employee compared to having a degree among women, whereas lower education was associated with lower odds of being an employee compared to having a degree among men. For both men and women, being African was associated with higher odds of being an employee compared to being White British, while being Pakistani was associated with lower odds compared to the reference group only for men. Lastly, the negative association between being a migrant (first‐ or second‐generation) and being an employee is only statistically significant among men.

**Table 4:** Odds ratios of being an employee

		Both genders	Men	Women
	*% of individuals in each group*	*Odds ratios*	*Odds ratios*	*Odds ratios*
Employee (N = 18,915)	86.00%			
Self‐employed (N = 2,965)	14.00%			
**Age**				
25–29 (ref)	12.40%	1	1	1
30–34	13.90%	0.79*	0.66***	1.07
35–39	15.40%	0.59***	0.55***	0.65**
40–44	16.40%	0.57***	0.52***	0.65**
45–49	15.30%	0.52***	0.47***	0.57***
50–54	12.90%	0.50***	0.43***	0.61**
55–59	10.10%	0.43***	0.39***	0.49***
60–64	3.60%	0.36***	0.32***	–
**Gender**				
Male (ref)	48.80%	1	–	–
Female	51.20%	2.32***		
**Marital status**				
Married (ref)	59.90%	1	1	
Single never married	25.80%	1.03	0.97	1.18
Separated/divorced	13.10%	1.07	0.99	1.17
Widowed	1.10%	1.29	1.37	1.17
**Education**				
Degree (ref)	31.80%	1		
Other high	11.70%	1	0.92	1.2
A level	9.80%	0.92	0.81*	1.14
GCSE	22.90%	0.98	0.78***	1.46***
Other qualification	9.00%	0.97	0.73***	1.68***
No qualifications	14.70%	0.87*	0.66***	1.63***
**Housing tenure**				
Own outright (ref)	16.80%	1		
Own with mortgage	56.50%	1.14*	1.15*	1.1
Rented from local authority	6.30%	2.05***	1.92***	2.16***
Rented from Housing Association	4.20%	1.40**	1.35*	1.39
Rented from employer	1.20%	1.14	1.37	0.7
Rented privately	14.60%	1.15	1.1	1.19
Other	0.20%	1.3	1.22	1.41
**Ethnicity**				
White British (ref)	75.50%	1		
Polish	1.00%	0.93	1.07	0.8
Other White	4.40%	0.94	1.07	0.77
Mixed	1.50%	1.08	0.98	1.2
Indian	4.50%	1.34*	1.24	1.46
Pakistani	2.30%	0.70**	0.66*	0.96
Bangladeshi	1.60%	1	0.96	1.69
Other Asian	2.00%	1.15	1.19	1
Caribbean	2.40%	1.38	1.34	1.37
African	2.90%	1.71**	1.53*	1.91*
Other ethnic groups	1.70%	0.93	1.31	0.54*
**Migrant history**				
Not a migrant (ref)	74.80%	1		
2nd‐generation migrant	5.90%	0.79*	0.68**	1.02
1st‐generation migrant	19.10%	0.76*	0.74*	0.85
**Constant**		7.10***	9.50***	10.74***

*Notes*: N = 21,880, *** p < 0.001; ** p < 0.01; * p < 0.05.

## Association between Ethnicity and One's Chances of Working for an Employer with a Pension Scheme

Among all employees, table 5 shows the odds ratios of working for an employer who offers a pension scheme, and it is a combination of demographic and socio‐economic characteristics, including ethnicity, which explains one's odds of working for an employer who offers a pension scheme. Being aged between 30 and 34 or 50 and 54 years, compared to those aged 25–29, is positively associated with one's odds of working for an employer with a pension scheme, while being female and indicators of a lower socio‐economic status, such as education which is lower than a degree, renting one's home, belonging to the semi‐routine/routine/never worked/unemployed social classifications and to the lower quintiles of the take‐home pay distribution, are negatively associated with such odds. Coming from an ethnic minority group was negatively associated with working for an employer with a pension scheme compared to the White British group, and this effect is statistically significant for the Other White, Indian, Pakistani, Bangladeshi, Other Asian and Other ethnic groups. Second‐generation migrants are more likely to work for an employer with a pension scheme than non‐migrants. Once gender was taken into account in Models 2 and 3, the results remained broadly similar, with a few exceptions. The negative impact of coming from the Polish group on one's odds of working for an employer with a pension scheme is observed among women, but was not statistically significant for men, and the same was true for the (negative) impact of belonging to a lower social classification. Lastly, the positive association between being a second‐generation migrant and working for an employer with a pension scheme is statistically significant for women only, whereas in the men's model, being a first‐generation migrant is negatively associated with working for an employer offering a pension scheme, compared to non‐migrants.

**Table 5:** Odds ratios of working for an employer who offers a pension scheme

		Both genders	Men	Women
	*% of individuals in each group*	*Odds ratios*	*Odds ratios*	*Odds ratios*
Working for employer with pension scheme (N = 13,408)	72.30%			
Not working for employer with pension scheme (N = 5,145)	27.70%			
**Age**				
25–29 (ref)	12.90%	1	1	1
30–34	14.30%	1.21**	1.08	1.33**
35–39	15.50%	1.07	1.06	1.09
40–44	16.50%	1.12	1.08	1.16
45–49	15.30%	1.14	1.05	1.25*
50–54	12.80%	1.23*	1.17	1.30*
55–59	9.70%	1.07	1.06	1.08
60–64	3.00%	1.2	1.08	
**Gender**				
Male (ref)	45.90%	1		
Female	54.10%	0.90*		
**Marital status**				
Married (ref)	59.20%	1	1	1
Single never married	26.40%	0.95	0.92	0.96
Separated/divorced	13.30%	1	0.94	1.03
Widowed	1.10%	1.19	1.51	1.08
**Education**				
Degree (ref)	32.20%	1	1	1
Other high	12.00%	0.85*	1	0.78**
A level	9.70%	0.92	0.98	0.87
GCSE	23.20%	0.86*	0.79**	0.92
Other qualification	8.80%	0.91	0.80*	0.99
No qualifications	14.00%	0.71***	0.61***	0.81*
**Housing tenure**				
Own outright (ref)	16.10%	1	1	1
Own with mortgage	57.00%	1.05	0.94	1.15
Rented from local authority	6.60%	0.69***	0.62***	0.77*
Rented from Housing Association	4.20%	0.72**	0.67*	0.76*
Rented from employer	1.20%	0.68*	0.78	0.55*
Rented privately	14.60%	0.71***	0.65***	0.75**
Other	0.20%	0.75	1	0.54
**Ethnicity**				
White British (ref)	76.20%	1	1	1
Polish	0.90%	0.89	1.45	0.51*
Other White	4.30%	0.73**	0.81	0.62**
Mixed	1.60%	0.77	0.75	0.75
Indian	4.50%	0.57***	0.66*	0.49***
Pakistani	2.00%	0.56***	0.57**	0.58*
Bangladeshi	1.50%	0.38***	0.32***	0.63
Other Asian	2.00%	0.69*	0.74	0.62*
Caribbean	2.40%	0.79	0.7	0.81
African	3.00%	0.83	0.93	0.74
Other ethnic groups	1.70%	0.63**	0.72	0.55*
**Occupational social class**				
Management and professional (ref)	47.50%	1	1	1
Intermediate	15.80%	1.04	1.13	0.96
Lower supervisory and technical	8.40%	0.9	1.02	0.81
Semi‐routine, routine and never worked/long‐term unemployed	28.30%	0.84**	1.03	0.70***
**Migrant history**				
Not a migrant (ref)	75.50%	1	1	1
2nd‐generation migrant	5.90%	1.45**	1.34	1.54**
1st‐generation migrant	18.50%	0.85	0.69**	1.06
**Quintile of take‐home pay at last payment**				
Fifth highest (ref)	18.00%	1	1	1
Missing	13.00%	0.33***	0.38***	0.24***
Fourth	16.40%	0.88	0.97	0.68**
Third	19.30%	0.63***	0.68***	0.50***
Second	17.00%	0.33***	0.30***	0.29***
First lowest	16.20%	0.13***	0.15***	0.09***
**Occupational sector**				
Private (ref)	60.40%	1	1	1
Public	39.60%	6.38***	5.61***	6.83***
**Constant**		5.28***	5.66***	5.42***

*Notes*: N = 18,553, *** p < 0.001; ** p < 0.01; * p < 0.05.

## Association between Ethnicity and One's Chances of Being a Member of their Employer's Pension Scheme

Table 6 shows the odds ratios of belonging to an employer's pension scheme among those employees who worked for an employer who offered such a scheme. Older age and being married are associated strongly with the odds of belonging to one's employer's pension scheme, and although gender was not statistically significant in this model nevertheless the impact of all demographic and socio‐economic determinants is quite similar for men and women when the analysis is run separately (Models 2 and 3). As before, indicators of an individual's lower socio‐economic status, such as lower education, renting one's home, belonging to the lower social classifications and belonging to the lower quintiles of the take‐home pay distribution, are negatively associated with being a member of one's employer's pension scheme. The effect of ethnicity in this step of the analysis is negligible apart from Pakistani and Bangladeshi individuals whose odds of being members of their employer's pension scheme were 0.61 and 0.59, respectively, compared with the odds among British White individuals, and these two groups' lower odds were reflected among women separately but not among men, Lastly, being a migrant (first‐ or second‐generation) is negatively associated with belonging to the employer's pension scheme compared to being a non‐migrant, and this is statistically significant for first‐generation migrants among men separately.

**Table 6:** Odds ratios of being a member of one's employer's pension scheme

		Both genders	Men	Women
	*% of individuals in each group*	*Odds ratios*	*Odds ratios*	*Odds ratios*
Being a member (N = 9,926)	74.10%			
Not being a member (N = 3,473)	25.90%			
**Age**				
25–29 (ref)	11.90%	1	1	1
30–34	14.20%	1.29**	1.16	1.38**
35–39	15.40%	1.61***	1.59***	1.60***
40–44	16.70%	2.07***	2.22***	1.94***
45–49	15.80%	2.35***	2.93***	2.03***
50–54	13.30%	2.44***	2.80***	2.19***
55–59	9.80%	2.12***	2.34***	1.93***
60–64	3.00%	1.79***	1.77***	–
**Gender**				
Male (ref)	45.00%	1		
Female	55.00%	0.92		
**Marital status**				
Married (ref)	60.10%	1	1	1
Single never married	25.60%	0.86*	0.88	0.86*
Separated/divorced	13.10%	0.76***	0.68***	0.80*
Widowed	1.10%	1.07	0.7	1.47
**Education**				
Degree (ref)	36.40%	1	1	1
Other high	12.80%	0.73***	0.77*	0.70***
A level	9.80%	0.83*	0.77*	0.92
GCSE	22.20%	0.75***	0.79*	0.72***
Other qualification	8.10%	0.82*	0.93	0.75*
No qualifications	10.80%	0.75***	0.83	0.70**
**Housing tenure**				
Own outright (ref)	16.60%	1	1	1
Own with mortgage	61.30%	1.02	0.95	1.05
Rented from local authority	5.00%	0.56***	0.59**	0.52***
Rented from Housing Association	3.50%	0.53***	0.52***	0.53***
Rented from employer	1.10%	1.03	1.53	0.59
Rented privately	12.20%	0.57***	0.53***	0.60***
Other	0.20%	0.62	0.78	0.54
**Ethnicity**				
White British (ref)	79.00%	1	1	1
Polish	0.60%	0.8	0.94	0.64
Other White	4.10%	0.96	0.91	0.96
Mixed	1.60%	0.91	0.79	0.95
Indian	3.80%	0.89	0.95	0.85
Pakistani	1.60%	0.61**	0.72	0.55*
Bangladeshi	1.00%	0.59*	0.99	0.35**
Other Asian	1.70%	0.86	0.99	0.76
Caribbean	2.50%	1.27	1.25	1.22
African	2.70%	0.85	0.87	0.86
Other ethnic groups	1.40%	0.78	0.85	0.74
**Occupational social class**				
Management and professional (ref)	54.20%	1	1	1
Intermediate	16.80%	1.02	0.95	1.06
Lower supervisory and technical	7.20%	0.77**	0.77*	0.76
Semi‐routine, routine and never worked/long‐term unemployed	21.80%	0.57***	0.58***	0.58***
**Migrant history**				
Not a migrant (ref)	78.20%	1	1	1
2nd‐generation migrant	6.50%	0.75*	0.71	0.79
1st‐generation migrant	15.30%	0.74**	0.66*	0.8
**Quintile of take‐home pay at last payment**				
Fifth highest (ref)	22.00%	1	1	1
Missing	12.00%	0.45***	0.48***	0.40***
Fourth	19.90%	0.60***	0.58***	0.62***
Third	21.70%	0.40***	0.34***	0.42***
Second	15.70%	0.27***	0.21***	0.29***
First lowest	8.70%	0.25***	0.34***	0.19***
**Occupational sector**				
Private (ref)	49.80%	1	1	1
Public	50.20%	3.13***	3.06***	3.25***
Constant		4.47***	4.52***	4.13***

*Notes*: N = 13,399, *** p < 0.001; ** p < 0.01; * p < 0.05.

## Discussion and Conclusions

In this article, we investigate the association between ethnicity and an individual's chances of:

1. being in paid work;
2. working as an employee;
3. working for an employer who offers a pension scheme; and
4. being a member of an employer's pension scheme.

This article lends support to existing findings from earlier research which point to significant differentials between ethnic minorities and the White British population in terms of pension protection (Ginn and Arber 2001). Unlike existing research in this area, the analysis presented in this article takes into account the impact of the occupational sector in which an individual works, as well as that person's migration history, and in addition presents evidence regarding the pension protection of the relatively ‘new’ minority of Polish individuals. The data presented provides strong empirical evidence that minority ethnicity has a differential and negative association for different ethnic groups in the first three areas (being in paid work; being an employee; working for an employer with a pension scheme). However, ethnicity shows a significant association with one's chances of being a member of an employer's scheme only for Pakistani and Bangladeshi individuals. Such individuals were significantly less likely to be members of an employer's scheme than White British individuals, however no significant differences were found for all the other ethnic groups.

The results of this analysis are broadly compatible with existing research which points to minority ethnic groups, and particularly the Pakistani and Bangladeshi groups within the BME population as a whole, as being disadvantaged compared to the White British majority in terms of their pension protection by being less likely to participate in the labour market (Allmark *et al*. 2010) and being more likely to be self‐employed (PPI 2003).

The strong effect of indicators reflecting higher socio‐economic status on one's odds of being employed, being an employee, working for an employer who offers a pension scheme and being a member of that scheme, also lends support to existing literature. For example, Bryan *et al*. (2011) analyzed data from the Wealth and Assets Survey and found that saving among employees is associated with older age, high education, White ethnicity and home ownership. The positive impact of older age on the odds of being a member of one's employer's pension scheme may indicate one's increasing capacity to contribute to an occupational pension scheme as one's salary increases over one's working life. This finding may also indicate a significant cohort effect, with those in the latter part of their working life having benefited the most from the development of defined benefit pension schemes, while younger cohorts of working age men and women experience a decline in the availability of an occupational pension scheme. Indeed, the private sector saw a fall in active membership in defined benefit schemes from 3 million in 2006 to 1.9 million in 2011, while membership in defined contribution schemes remained stable during that time (at around 1 million) (ONS 2012b). These absolute numbers represent a drop over time in the share of employees' membership of private sector defined benefit schemes from about one‐third of all employees in 1997 to 9 per cent in 2001 (ONS 2012c). The results also show that women are less likely than men to work for an employer offering a pension scheme, although their lower likelihood to be a member of such a scheme was not statistically significant. This result tallies with existing research noting that gender *per se* does not appear to have an effect on one's likelihood of saving for retirement, whereas age and social class might have a stronger effect in this respect (Adami and Gough 2008). However, there is evidence in the literature that women's occupational pension membership has been increasing steadily since the late 1990s, while men's has been decreasing, thereby closing the gender gap in this respect (ONS 2012c). There are differentials between men and women in the likelihood of being employed, but once working for an employer who offers a pension, gender is not a statistically significant factor in one's chances of being a member of such a scheme.

The results in this article suggest that being in paid work is a crucial step towards better pension prospects for individuals from BME groups, but that this is not enough. Indeed, the article shows that Polish individuals are the only ethnic group who are more likely than the White British to be in paid work, however their odds of being employees, or working for an employer who offers a pension scheme, or being a member of their employer's pension scheme are similarly low to other ethnic groups and lower than those of the White British group. Working as an employee, rather than as self‐employed, and working for an employer who offers a pension scheme, are fundamental facilitators of a working‐age individual's pension prospects. Government policy, which aims to roll out the auto‐enrolment scheme to smaller companies by 2017, will go some way towards ensuring that future cohorts of individuals from BME groups enter retirement with stronger pension protection. However, an important caveat to these reforms relates to the potential risk attached to defined‐contribution pension schemes offered through auto‐enrolment, relative to the protection provided by continuously decreasing defined‐benefit schemes (Price 2012). In addition, facilitating the pension protection of self‐employed workers should also be part of the future policy agenda. It is striking that ethnic inequalities have not narrowed over time, highlighting that such proactive policies to address the employment and pensions gap are required.

## Notes

1 The category of ‘Other ethnic groups’ includes: ‘Any other black background’, ‘Arab’ and ‘Any other ethnic group’.

2 In this variable, individuals who have no qualifications but have vocational qualifications have been included into different categories of highest educational qualification by the UKHLS dataset. ‘HNC/HND’ is coded as ‘Other higher qualification’; ‘Modern apprenticeship/trade apprenticeship’, ‘NVQ/SVQ – Level 3–5’, ‘ONC/OND’, ‘BTEC/BEC/TEC/EdExcel/LQL’, and ‘SCOTVEC, SCOTEC or SCOTBEC’ are coded as ‘A‐levels’; ‘GNVQ/GSVQ’, ‘NVQ/SVQ – Level 1–2’ are coded as ‘GCSE’; ‘Youth training certificate’, ‘Key Skills’, ‘Basic skills’ ‘Entry level qualifications (Wales)’, ‘RSA/OCR/Clerical and commercial qualifications (e.g. typing/shorthand/book‐keeping/commerce)’, ‘Other vocational, technical or professional qualification’ and ‘City and Guilds Certificate’ are coded as ‘Other qualification’.
